# Interplay between cellular changes in the knee joint, circulating lipids and pain behaviours in a slowly progressing murine model of osteoarthritis

**DOI:** 10.1002/ejp.2036

**Published:** 2022-09-19

**Authors:** Peter R. W. Gowler, James Turnbull, Mohsen Shahtaheri, David A. Walsh, David A. Barrett, Victoria Chapman

**Affiliations:** ^1^ Pain Centre Versus Arthritis and NIHR Nottingham Biomedical Research Centre, School of Life Sciences University of Nottingham Nottingham UK; ^2^ Centre for Analytical Bioscience, Advanced Materials and Healthcare Technologies Division, School of Pharmacy University of Nottingham Nottingham UK; ^3^ Pain Centre Versus Arthritis and NIHR Nottingham Biomedical Research Centre, School of Medicine University of Nottingham Nottingham UK

## Abstract

**Background:**

Synovial inflammation has known contributions to chronic osteoarthritis (OA) pain, but the potential role in transitions from early to late stages of OA pain is unclear.

**Methods:**

The slowly progressing surgical destabilization of the medial meniscus (DMM) murine OA model and sham control, was used in male C57BL/6J mice to investigate the interplay between knee inflammation, plasma pro‐ and anti‐inflammatory oxylipins and pain responses during OA progression. Changes in joint histology, macrophage infiltration, chemokine receptor CX3CR1 expression, weight bearing asymmetry, and paw withdrawal thresholds were quantified 4, 8 and 16 weeks after surgery. Plasma levels of multiple bioactive lipid mediators were quantified using liquid chromatography with tandem mass‐spectrometry (LC–MS/MS).

**Results:**

Structural joint damage was evident at 8 weeks post‐DMM surgery onwards. At 16 weeks post‐DMM surgery, synovial scores, numbers of CD68 and CD206 positive macrophages and pain responses were significantly increased. Plasma levels of oxylipins were negatively correlated with joint damage and synovitis scores at 4 and 8 weeks post‐DMM surgery. Higher circulating levels of the pro‐resolving oxylipin pre‐cursor 17‐HDHA were associated with lower weight bearing asymmetry at week 16.

**Conclusions:**

The transition to chronic OA pathology and pain is likely influenced by both joint inflammation and plasma oxylipin mediators of inflammation and levels of pro‐resolution molecules.

**Significance:**

Using a slow progressing surgical model of osteoarthritis we show how the changing balance between local and systemic inflammation may be of importance in the progression of pain behaviours during the transition to chronic osteoarthritis pain.

## 1 INTRODUCTION

Osteoarthritis (OA) is a whole‐organ disorder affecting synovial joints, with the most significant symptom being chronic pain (Neogi, 2013). OA is associated with synovial inflammation, which may contribute both to joint pain and pathology (Baker et al., 2010; Guermazi et al., 2014). Inflammation causes pain by activating and sensitizing peripheral nociceptors (Matsuda et al., 2019). Clinical efficacy of treatments that suppress inflammation or sensitisation, including intra‐articular glucocorticosteroid administration (Bellamy et al., 2006; Osani et al., 2019) and nerve growth factor (NGF)‐blocking antibodies (Lane et al., 2010), indicate key roles for inflammation and peripheral sensitisation in OA pain.

The earliest stages of human OA are a challenge to investigate due to the slow rate of progression (over many years) and the lack of a clearly defined onset (Luyten et al., 2018). ‘Early OA’ is often defined structurally (Luyten et al., 2018), although biochemical changes are demonstrable within structurally normal cartilage from OA joints (Qazi et al., 2007), and patients may have pain before structural changes become apparent (Wang et al., 2018). OA structural changes progress rapidly in most rodent models. Destabilization of the medial meniscus (DMM) murine model of OA progresses relatively slowly over the course of weeks rather than days (Glasson et al., 2007; Gowler, Mapp, et al., 2020), allowing mechanistic investigation of the events underpinning the transition from the early to late stages of OA pathology and pain.

Inflammation may be an early feature of human OA (Smith et al., 1997). Joint inflammation is mediated by bioactive lipids, cytokines, chemokines, damage‐associated molecular patterns (DAMPs) and growth factors (Jónasdóttir et al., 2017; Liu‐Bryan & Terkeltaub, 2012). The presence of these mediators in OA is associated with cellular infiltration and proliferation in the synovium, including increased numbers of macrophages (Pessler et al., 2008). Synovial macrophage activation is associated with pain in people with OA (Kraus et al., 2016).

Macrophages and their mediators facilitate inflammation and peripheral pain sensitisation but also contribute to the resolution of inflammation (Buckley et al., 2014). Resolution is an active process driven by pro‐resolution pathways, of which specialized pro‐resolving lipid mediators (SPMs) are key signalling molecules (Buckley et al., 2014). Broadly, macrophages can have either pro‐ (M1) or anti‐inflammatory (M2) phenotypes (Yunna et al., 2020) and macrophage subtype distribution changes following injury through to repair (Dalli & Serhan, 2017). The chemokine receptor CX3CR1 stimulates leukocyte migration and is expressed in a subset of ‘membrane’ forming macrophages (Culemann et al., 2019; Klosowska et al., 2009). CX3CR1‐positive macrophages form a protective lining to the synovium which restricts inflammatory responses (Culemann et al., 2019). In inflammatory arthritis this protective function is impaired and CX3CR1‐positive cells undergo morphological changes (Culemann et al., 2019). We have also previously shown that the ligand for CX3CR1, CX3CL1 is correlated to histological inflammation in human OA synovia (Gowler, Li, et al., 2020).

Alongside inflammatory signalling in the diseased joint, circulating levels of pro‐ and anti‐inflammatory molecules are also altered in people with OA pain (Attur et al., 2015). These include lipid mediators derived from polyunsaturated omega‐3 (ω‐3) and omega‐6 (ω‐6) fatty acids which regulate the initiation, maintenance and resolution of inflammatory responses (Gilroy & Bishop‐Bailey, 2019). We have reported that high circulating levels of the ω‐3 derived 17‐hydroxydocasahexaenoic acid (17‐HDHA), a precursor to SPM D‐series resolvins, is associated with higher thermal pain thresholds in healthy volunteers and with lower pain in people with OA (Valdes et al., 2017). Serum levels of ω‐3‐derived lipids were associated with less severe OA pathology in a rodent model of OA (Wu et al., 2017).

Understanding how systemic factors may reflect local changes in the joint could help to identify clinically relevant markers of OA pain. Our aim, therefore, was to advance knowledge of the interplay between key events within the knee joint (histology, macrophage number CD68, CD206 [Sambamurthy et al., 2018] and expression of the chemokine receptor CX3CR1), levels of circulating pro‐ and anti‐inflammatory oxylipins and pain behaviour, over the development of the DMM model of OA pain.

## 2 METHODS

All the animal studies reported herein complied with the regulations set out by the Animal (Scientific Procedures) Act 1986, and all data are reported in accordance with the ARRIVE guidelines. Ten‐week‐old male C57BL/6J mice (weight: 21–27.4 g) (*n* = 60) were used. Mice were group‐housed in environmentally enriched individually ventilated cages in temperature controlled (23°C) rooms with access to food and water ad libitum. The experimenter was blinded to the experimental conditions of the animals throughout the study and animals were allocated randomly to groups by a third party.

### 2.1 Behavioural testing

Mice were anaesthetised with 2% isoflurane (carried by 1 L/min 100% O_2_) before undergoing either DMM (*n* = 30) or sham (*n* = 30) surgery. Once mice were areflexic, DMM mice had the medial meniscotibial ligament (MMTL) transected to destabilize the medial meniscus (Glasson et al., 2007). The sham surgery was the same as the DMM, apart from the transection of the MMTL.

Mice were tested for weight‐bearing asymmetry and ipsilateral hind‐paw withdrawals before the surgery and then once a week up to 16 weeks post‐surgery. Mice were habituated to the behavioural tests for 1 week prior to the start of the study, and before each testing session mice were given 30 min to acclimatize to the testing environment. Weight‐bearing asymmetry was measured using an incapacitance meter (Linton Instruments) with three measurements of 3 s taken per animal per time‐point (Gowler, Mapp, et al., 2020). Hind paw withdrawal thresholds (PWTs) were measured using calibrated von Frey monofilaments. Each hind paw was stimulated five times by each hair (0.07–2 g) in ascending order, with the percentage of nocifensive responses recorded. The 50% withdrawal threshold was measured using the EC50 of the log transformed responses to this battery of hairs (Hulse et al., 2014). Studies were terminated at three different time‐points, 4 weeks (sham *n* = 10, DMM *n* = 10), 8 weeks (sham *n* = 10, DMM *n* = 10) and 16 weeks post‐surgery (sham *n* = 10, DMM *n* = 10). Group sizes were powered to detect a difference in weight‐bearing asymmetry between DMM mice and sham mice at 16 weeks postsurgery based on previous data in this model. Separate cohorts of mice were euthanised by intra‐peritoneal injection of sodium pentobarbital before blood was collected and plasma prepared for LC/MS–MS analysis and knee joints were collected for histological analysis.

### 2.2 Joint histology

Knee joints were dissected postmortem and fixed in 10% neutral buffered formalin for 48 h, and then decalcified in a 10% EDTA (with 7.5% PVP) solution for 10 days before being processed with xylene and embedded in paraffin. Sagittal sections (5 μm) across the depth of the medial side of the joint were collected. Three sections per joint were then stained for haematoxylin and eosin (H&E) and three sections per joint were then stained for safranin O (Saf‐O) as previously described (Ashraf et al., 2019). Slides were blinded and then joint pathology was assessed by two independent scorers based on previously published scoring criteria (Supplementary Table S1; Ashraf et al., 2019).

### 2.3 Fluorescent labelling of CD68 and CD206 in the synovia

The numbers of CD68 (a pan‐macrophage marker) positive macrophages and CD206 (a marker of the more anti‐inflammatory M2‐like macrophages, Yunna et al., 2020) positive macrophages in the synovia were quantified by analysis of macrophage fractional area. Due to the limited size of the medial aspect of the knee in mice we used two sagittal sections per mouse per marker to enable us to quantify multiple markers in these experiments. Sections underwent antigen retrieval in a pH 9 Tris EDTA buffer for 20 min at 60°C, sections were then washed in 0.1 M PBS before being incubated in a blocking solution for an hour (5% serum, 0.5% Triton‐X100). Sections were then either incubated in 1 μg/100 μl rat anti‐CD68 (Bio‐Rad: MCA1957) or 1 μg/200 μl rabbit anti‐CD206 (Abcam: ab64693) overnight at room temperature (RT). Sections were then washed before being developed with either 1 μg/200 μl Alexafluor 594 conjugated donkey anti‐rat secondary antibody (Invitrogen: A21209) or 1 μg/200 μl Alexafluor 488 conjugated goat anti‐rabbit (Invitrogen: A11008) secondary antibody for 2 h at RT. Sections were incubated with 1:1000 DAPI for 20 mins at RT to counterstain nuclei.

Images were taken with a Leica 200 M microscope using a 40 × 1.3 objective. Two fields of view were taken for each section of the synovia from the anterior and posterior portions of the medial tibial plateau. Images were auto‐thresholded using the Huang method and the fractional area of suprathreshold areas of labelling in the synovial sections were quantified using ImageJ software (Gowler, Li, et al., 2020).

### 2.4 Quantification of synovial CX3CR1

Expression of CX3CR1, a chemokine receptor which stimulates leukocyte migration (Klosowska et al., 2009), was quantified in two sagittal sections per mouse. Sections underwent antigen retrieval at 60°C in a pH 9 Tris EDTA buffer for 20 min. Endogenous peroxidase was inhibited by incubation with BLOXALL (Vector Laboratories). Sections were incubated in a blocking solution for an hour at RT (5% serum, 0.5% Triton‐X100), and then incubated with 1:75 rabbit anti‐CX3CR1 (Abcam: ab8021) for 48 h at RT. Labelling was developed using a Universal kit (Vector Laboratories: PK‐6200). Sections were incubated with DAB HRP substrate (Vector Laboratories: SK‐4100) for 7 min, before being counterstained with Harris haematoxylin. Images were taken using a Zeiss Axioskop 50 microscope. Two blinded observers counted the number of positively labelled cells and the average cell counts were taken and normalized to synovia area.

### 2.5 Liquid chromatography with tandem mass spectrometry

Targeted LC–MS/MS analysis was carried out on plasma samples collected post‐mortem from DMM and sham mice at 4‐, 8‐ and 16‐weeks post‐surgery (*n* = 10 per group). The method was adapted from that previously reported (Wong et al., 2014) for the analysis of 33 bioactive lipids including members of the specialized pro‐resolution mediators (SPMs); resolvin D5 (RvD5), maresin 2, the D series resolvin precursors 17‐hydroxydocosahexaenoic acid (17‐HDHA). Briefly, serum samples were processed with solvent extraction and solid‐phase extraction, and LC–MS/MS used negative ionization mode and quantification was performed using the analyte to internal standard peak area ratio against a fully extracted calibration line. Endogenous (n = 6) and spiked QC standards (*n* = 6) were extracted and analysed across the sample run and met QC acceptance criteria of <15%CV.

### 2.6 Data analysis

Data were analysed with GraphPad Prism (V. 7). All data were tested for normality using the D'Agostini Pearson normality test and tests selected accordingly. Grubb's outliers test was used to identify outliers. Differences in behavioural data and, immunohistochemistry between groups were analysed by two‐way ANOVA with Bonferroni corrected multiple comparisons. Differences in histopathology between groups were analysed by Kruskal Wallis with Dunn's corrected multiple comparisons. The potential relationship between plasma levels of lipid mediators and pain scores, pathology and markers of inflammatory cells were analysed by Spearman's Rho. Data are presented as mean ± SEM.

## 3 RESULTS

### 3.1 Timeline of structural joint pathology in the destabilization of the medial meniscus model

**FIGURE 1:**
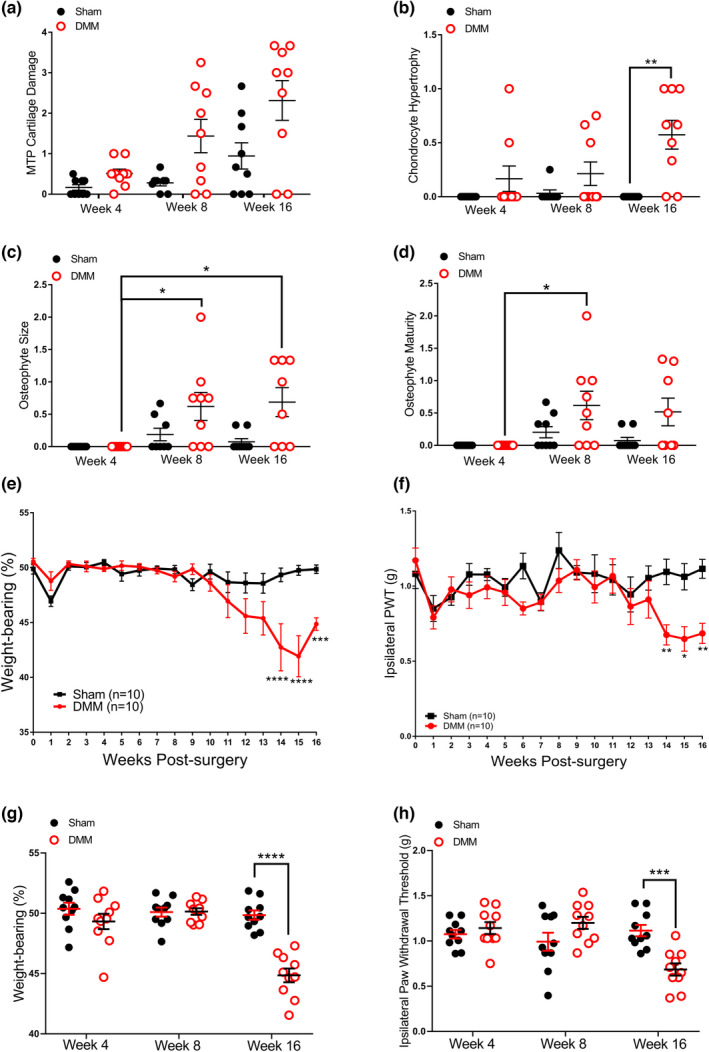
Adult C57BL6 mice underwent either DMM (*n* = 30) or sham surgery (*n* = 30). Quantification of medial tibial plateau cartilage damage (a), chondrocyte hypertrophy (b), osteophyte size (c), and osteophyte maturity (d) in mice at 4 weeks, 8 weeks and 16 weeks post‐DMM and sham surgery. Data analysed by Kruskal–Wallis with Dunn's multiple comparisons. *p < 0.05, **p < 0.01, ***p < 0.001. The time‐courses of both weight‐bearing asymmetry (e) and ipsilateral hind‐paw withdrawal thresholds (f) in the 16‐week post‐surgery groups are shown. Data analysed by two‐way ANOVA with Bonferroni's multiple comparisons. *p < 0.05, **p < 0.01, ***p < 0.001, ****p < 0.0001 DMM vs sham. Weight‐bearing asymmetry (g) and ipsilateral hind‐paw withdrawal thresholds (h) were assessed at 4, 8 and 16 weeks post‐surgery. Data analysed by two‐way ANOVA with Bonferroni's multiple comparisons. MTP, Medial tibial plateau.

Knee joint structural integrity was compared between time‐matched DMM‐operated and sham‐operated groups (Supplementary Figure S1). Changes in joint structure, consistent with OA, were observed in DMM‐operated mice from 8 weeks post‐surgery. Analysis by Kruskal‐Wallis showed a significant difference in articular cartilage degradation between all groups (*p* = 0.01). While cartilage degradation was elevated in DMM mice from week 8 pairwise comparisons showed no significant difference between DMM and sham at any time‐point (Figure 1a). Chondrocyte hypertrophy scores were higher than those for sham controls at 16 weeks post‐surgery (*p* = 0.001; Figure 1b). Osteophytes were observed in some DMM‐operated mice from 8 weeks, with osteophyte size in DMM‐operated mice being significantly increased at week 8 (*p* = 0.02) and week 16 (*p* = 0.02) compared with week 4. While osteophyte maturity was significantly higher in mice 8 weeks post‐DMM compared with mice 4 weeks post‐DMM (Figure 1c,d).

### 3.2 Co‐occurrence of synovial inflammation and onset of pain behaviour in the destabilization of the medial meniscus model

**FIGURE 2:**
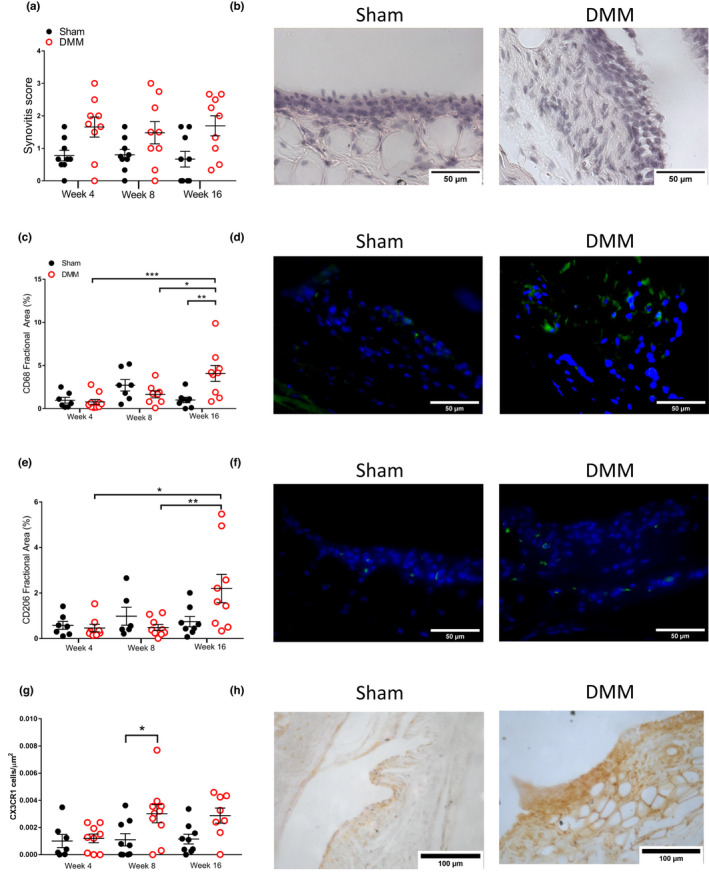
Quantification of synovitis score in mice 4, 8 or 16 weeks post‐DMM or sham surgery (a). Representative images of synovial hyperplasia in mice 16 weeks post‐sham or DMM surgery (b). Data analysed by Kruskal‐Wallis with Dunn's multiple comparisons. Quantification of CD68 fractional area between DMM‐ and sham‐operated mice at 4, 8 and 16 weeks post‐surgery (c). Representative images of synovial CD68 in mice 16 weeks post‐sham or DMM surgery (d). Quantification of CD206 fractional area between DMM‐ and sham‐operated mice at 4 weeks, 8 weeks and 16 weeks post‐surgery (e). Representative images of synovial CD206 in mice 16 weeks post‐sham or DMM surgery (f). Data analysed by two‐way ANOVA with Bonferroni's multiple comparisons. *p < 0.05, **p < 0.01. Quantification of CX3CR1 cell numbers normalized to area between DMM‐ and sham‐operated mice at 4, 8 and 16 weeks post‐surgery (g) data analysed by two‐way ANOVA with Bonferroni's multiple comparisons. *p < 0.05. Representative images of synovial CX3CR1 in mice 8 weeks post‐sham or DMM surgery (h).

Clinically, synovial inflammation is associated with OA pain. Following the initial post‐surgical period, significant changes in pain behaviours (weight‐bearing asymmetry and ipsilateral PWTs) were observed in the cohort taken out to 16 weeks post‐surgery (Figure 1e–h). There was greater weight‐bearing asymmetry exhibited by DMM mice, compared with sham mice, from 14 weeks post‐surgery (*p* < 0.0001), persisting at the end of the study at 16 weeks post‐surgery (*p* = 0.0005; Figure 1e). Similarly, there was a decrease in ipsilateral paw‐withdrawal thresholds in DMM mice compared with sham controls from 14 weeks post‐surgery (*p* = 0.009), which also persisted at 16 weeks post‐surgery (*p* = 0.0065; Figure 1f). Comparison of pain behaviours in the cohorts of mice euthanised at each time‐point confirmed that there were significant decreases in weight‐bearing asymmetry (*p* < 0.0001) and ipsilateral PWTs (*p* = 0.0002) at 16 weeks, but not at the other time‐points (Figures 1g,h). Synovitis scores were elevated in DMM mice compared with sham controls with a Kruskal–Wallis test showing a significant difference between all groups (*p* = 0.04); however, there was no significant differences at individual time‐points following pairwise comparisons (Figures 2a). Labelling of CD68 positive macrophages in the synovia was significantly increased at 16 weeks in the DMM mice, compared with sham mice (*p* = 0.003; Figures 2b,c). In addition, a marker of M2 polarized macrophages CD206 in synovia was significantly increased at 16 weeks in the DMM mice, compared with groups that were euthanised at 8 week (*p* = 0.01) and 4 weeks (*p* = 0.012) post‐surgery (Figure 2d,e). To potentially back‐translate our previous clinical finding of the correlation between CX3CL1 and synovial inflammation (Gowler, Li, et al., 2020), we also quantified CX3CR1‐immunoreactivity in the mouse synovium. CX3CR1 in the synovium was significantly elevated at 8 weeks in DMM mice, compared with sham mice (*p* = 0.02), although increases at 16 weeks were not statistically significant (Figure 2f).

### 3.3 Plasma levels of ω‐3/ω‐6 derived oxylipins are differentially associated with joint pathology and pain behaviour in the destabilization of the medial meniscus model of osteoarthritis

Plasma levels of 33 ω‐3/ω‐6 derived oxylipins were quantified at 4‐, 8‐ and 16‐weeks post‐surgery in the DMM model and sham controls (Supplementary Tables S2 and S3). We know from our clinical studies (Valdes et al., 2018) that the numbers of samples required to detect differences in levels of oxylipins between control and OA groups are far higher than achievable for experimental studies (Gowler et al., 2021). To limit the number of animals used, we therefore focused upon potential associations of oxylipins with joint pathology and pain behaviour.

#### 3.3.1 4 weeks post‐surgery

**FIGURE 3:**
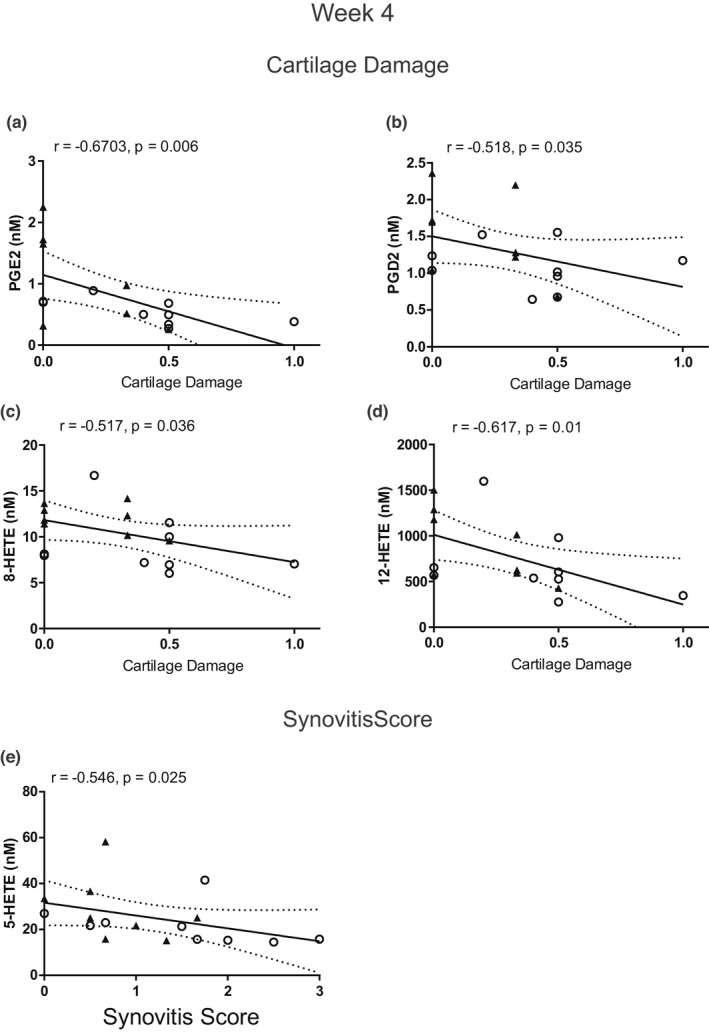
Correlation between cartilage damage and plasma concentrations of PGE2 (a), PGD2 (b), 8‐HETE (c) and 12‐HETE (d) in mice 4 weeks post‐surgery. Correlation between synovitis score and plasma concentrations of 5‐HETE (e)in mice 4 weeks post‐surgery. The open circles represent DMM‐operated mice, and the closed triangles represent sham‐operated mice. Data analysed by Spearman's Rho

Cartilage damage score was moderately negatively correlated with plasma concentrations of prostaglandin E2 (PGE2) (*r* = −0.67, *p* = 0.006), PGD2 (*r* = −0.52, *p* = 0.035), 8‐HETE (*r* = −0.52, *p* = 0.036) and 12‐HETE (*r* = −0.62, *p* = 0.01; Figure 3a–d). There was a moderate negative correlation of synovitis score with plasma 5‐hydroxyeicosatetraenoic acid (5‐HETE) (*r* = −0.55, *p* = 0.025; Figure 3e). There were no significant associations between plasma oxylipins and the macrophage markers CD68 and CD206 in the synovium, nor with pain behaviour, at this time‐point.

#### 3.3.2 8 weeks post‐surgery

**FIGURE 4:**
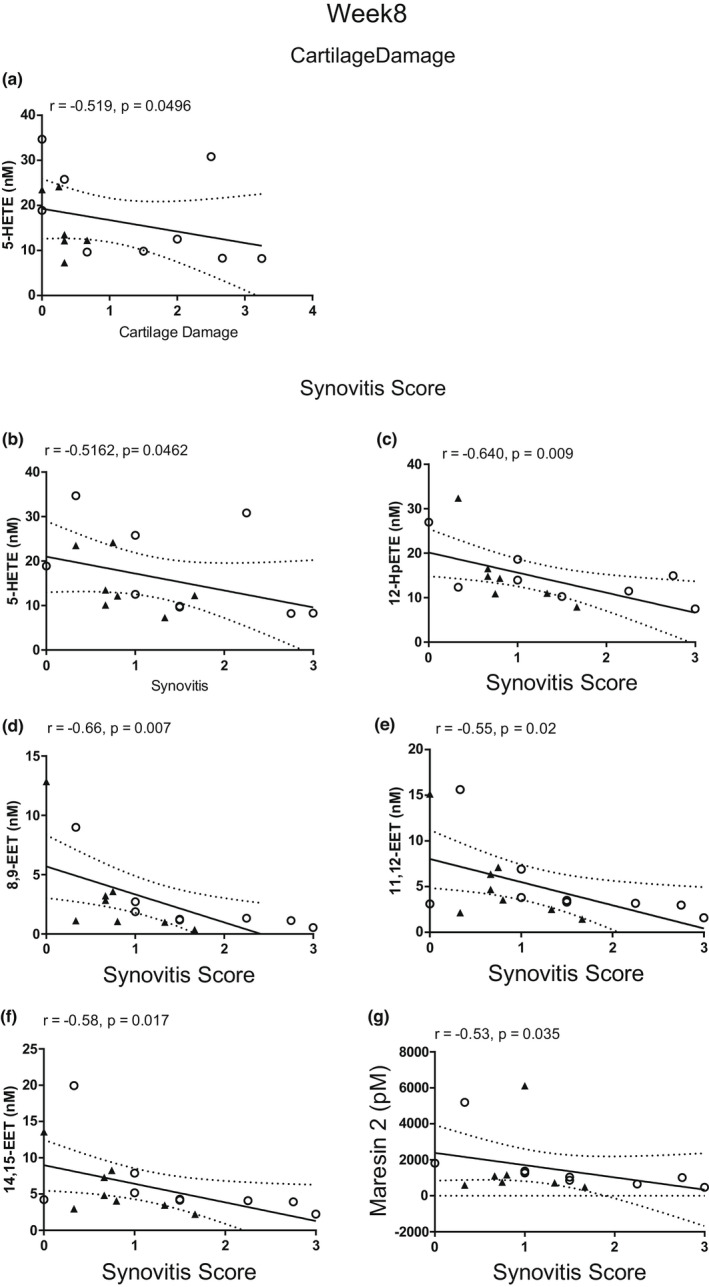
Correlation between cartilage damage and plasma concentrations of 5‐HETE (a) at 8 weeks post‐surgery. Correlation between synovitis score and plasma concentration of 5‐HETE (b), 12‐HpETE (c), 8,9‐EET (d), 11,12‐EET (e), 14,15‐EET (f) and Maresin 2 (g) in mice 8 weeks post‐surgery. The open circles represent DMM‐operated mice, and the closed triangles represent sham‐operated mice. Data analysed by Spearman's Rho.

Structural and inflammatory changes in the knee joint were associated with plasma levels of ω‐3/ω‐6 derived oxylipins. Plasma 5‐HETE was moderately negatively correlated with cartilage damage score (*r* = −0.52, *p* = 0.05; Figure 4a). There were significant moderate negative correlations between synovitis score and plasma levels of 5‐HETE (*r* = −0.52, *p* = 0.046), 12‐hydroperoxyeicosatraenoic acid (12‐HpETE) (*r* = −0.64, *p* = 0.009), maresin 2 (*r* = −0.53, *p* = 0.035), 8,9‐epoxyeicostraenoic acid (EET) (*r* = −0.66, *p* = 0.007), 11,12‐EET (*r* = −0.55, *p* = 0.02) and 14,15‐EET (*r* = −0.58, *p* = 0.017) (Figure 4b–g). There were no significant associations between plasma oxylipins and levels of the macrophage markers CD68 and CD206 in the synovium, nor with pain behaviour, at this time‐point.

#### 3.3.3 16 weeks post‐surgery

**FIGURE 5:**
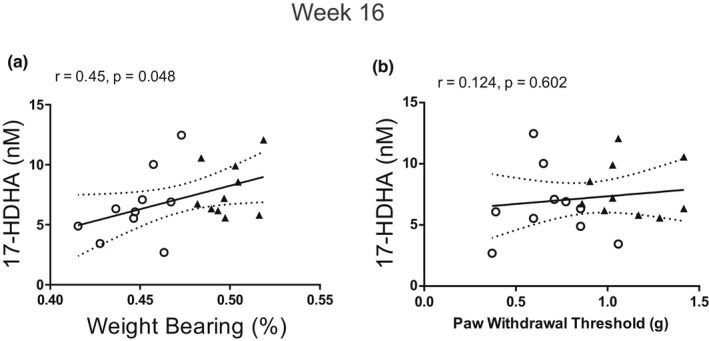
Correlation between weight‐bearing asymmetry and plasma concentration of 17‐HDHA (a) at 16 weeks post‐surgery. Correlation between ipsilateral paw withdrawal thresholds and plasma concentrations of 17‐HDHA (b) at 16 weeks post‐surgery. The open circles represent DMM‐operated mice and the triangles represent sham‐operated mice. Data analysed by Spearman's Rho

This was the first time‐point that pain behaviour was significant in the DMM model compared with sham controls (Figure 1g). There was a weak but significant positive correlation between higher plasma levels of 17‐HDHA with less weight bearing asymmetry, indicative of less pain behaviour (*r* = 0.45, *p* = 0.048; Figure 5a). There were no significant associations between plasma concentrations of 17‐HDHA and hind PWTs (Figure 5b). At this time‐point, 9‐oxo‐10E, 12Z‐octadecadienoic acid (9‐oxoODE) was moderately negatively correlated with cartilage damage (*r* = −0.69, *p* = 0.0045; Supplementary Figure S2). There were no significant associations between plasma oxylipins and the level of the macrophage markers CD68 and CD206 in the synovium.

## 4 DISCUSSION AND CONCLUSIONS

**FIGURE 6:**
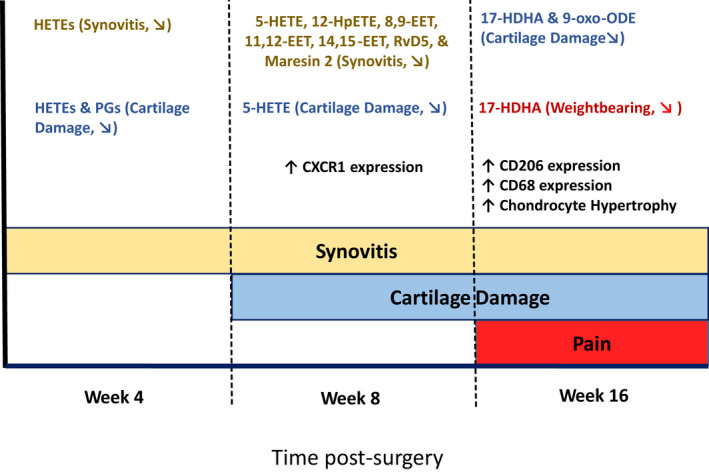
An illustration summarizing the changing relationships between circulating plasma lipids, joint pathology and pain behaviours in mice following DMM surgery. Synovial hyperplasia (yellow) was observed at all time‐points post‐surgery, cartilage damage (blue) was observed from 8 weeks post‐surgery, and pain behaviours (red) were observed from 16‐weeks post‐surgery. The text in the illustration indicates which oxylipins were correlated with joint pathology, and/or pain behaviours at each time‐point measured. The diagonal arrows indicate the direction of the correlation

We report an assessment of the complex changing picture of local and systemic inflammatory responses, joint pathology and pain behaviour in a murine model of OA (Figure 6). Sixteen weeks following DMM surgery, mice exhibited robust pain behaviour, including increased weight‐bearing asymmetry, compared with sham controls. Increased weight‐bearing asymmetry was significantly associated with lower plasma levels of the resolvin precursor 17‐HDHA. At this time‐point, levels of CD68 and CD206‐positive macrophages in the synovium were significantly increased compared with earlier time‐points. Additionally, plasma levels of 9‐OxoODE were negatively correlated with cartilage damage. At 8 weeks post‐DMM surgery, before pain behaviour was evident, levels of the anti‐inflammatory lipid mediators, the EETs and maresin‐2, were negatively correlated with synovial hyperplasia, suggesting a possible relationship between inflammatory events in the joint and systemic inflammatory responses. At this time‐point there was also an increase in the numbers of CX3CR1‐positive cells in the DMM synovium, compared with sham, possibly reflecting a role in driving later macrophage recruitment.

The slow progressing nature of the DMM model allowed the investigation of three distinct phases of the model; at 4 weeks post‐surgery when there is absence of pain behaviour and mild OA pathology, 8 weeks post‐surgery which also has absence of significant pain behaviour despite moderate OA pathology, and 16 weeks post‐surgery when both significant pain behaviour and pathology are evident. The development of joint pathology and pain behaviours observed following DMM surgery are consistent with previous findings. Mild cartilage damage has been reported from 4 weeks post‐DMM, which worsens over time (Botter et al., 2009; Glasson et al., 2007). We observed mild non‐significant changes in joint pathology at 4 weeks post‐surgery with more moderate pathology including chondrocyte hypertrophy, and osteophyte formation being observed at later time‐points. Increased weight‐bearing asymmetry and decreased ipsilateral hind‐paw withdrawals were evident from 11 weeks, as previously described (Driscoll et al., 2016; McNamee et al., 2010). It should be noted that mechanical allodynia as measured by hind‐paw withdrawals has been observed at earlier time‐points in some reports (Miller et al., 2012). These differences in the onset of mechanical allodynia may reflect differences in the testing paradigms used to ascertain mechanical withdrawal thresholds.

At 4‐ and 8‐weeks post‐DMM surgery, synovial hyperplasia and pain responses were not significant compared with the sham group. However, at 8 weeks, synovial expression of CD68‐ and CX3CR1‐positive cells was significantly increased in the DMM model compared with sham control. The chemokine receptor CX3CR1 has an established role in cellular migration (Klosowska et al., 2009) and is present on a subset of patrolling monocytes, which mediate early immune responses before differentiating into macrophages (Auffray et al., 2007). CX3CR1 is expressed by tissue‐resident macrophages forming the synovial lining under normal conditions, and these macrophages undergo morphological changes in inflammatory conditions (Culemann et al., 2019). Disruption of this barrier could increase the exposure of nociceptors in the synovia to pro‐sensitisation molecules present in synovial fluid. CX3CR1 has also been localized to multiple different cell types in the human rheumatoid arthritis synovia including macrophages, fibroblasts, dendritic cells and T cells (Blaschke et al., 2003). The presence of CX3CR1 on a variety of different cell types may explain why the significant increase in labelling was not concurrent with the increase in CD68 positive labelling at week 16. Our novel finding that CX3CR1‐positive cells are transiently increased in the synovium at 8 weeks following DMM surgery, may suggest a possible contribution to the onset of joint inflammation and pain responses at later time‐points.

At the latest time‐point studied, 16 weeks post‐DMM surgery, there was increased synovial expression of the pan‐macrophage marker CD68 (Yunna et al., 2020) compared with sham controls. Macrophages are established mediators of peripheral inflammation and nociception (Kiguchi et al., 2017). Their increased presence in the synovium is likely an important contributor to pain at this time‐point in the DMM model. Our data provide evidence that the DMM model mimics macrophage contributions to clinical OA pain (Kraus et al., 2016). Macrophages are readily polarized by their microenvironment to perform different functional roles (Murray, 2017). We, therefore, investigated the expression of the anti‐inflammatory/pro‐resolution M2 macrophage phenotype (CD206‐positive; Viola et al., 2019; Yunna et al., 2020). As previously reported (Utomo et al., 2020), synovial CD206 expression was not altered at 8 weeks post‐DMM surgery. Herein, we have extended the time frame studied, and at 16 weeks CD206 expression was increased in the DMM synovium, compared with 8 weeks post‐DMM, suggesting a switch to a pro‐resolution macrophage response at this time‐point. The increased synovial infiltration by macrophages at 16 weeks, may reflect an increased presence of anti‐inflammatory macrophages, coinciding with established pain behaviours.

We measured plasma concentrations of 33 oxylipins at each time‐point. As large numbers of mice per group are required to detect differences between the DMM and sham mice, coupled with the multiple time‐points studied, our analysis focused on potential associations between the plasma oxylipins, pain and joint pathology at the different time‐points. Members of the HETEs and PGs were negatively associated with cartilage damage at 4 weeks post‐surgery when OA pathology was mild, but at 8 weeks post‐surgery only 5‐HETE was still negatively associated with cartilage damage. The HETEs are produced from AA and have pro‐inflammatory roles (Powell & Rokach, 2015). Our data suggest HETEs may have protective roles in the pathogenesis of OA and/or may be an early biomarker of cartilage damage. At 4 weeks post‐surgery, lower levels of plasma PGs were also associated with greater cartilage damage. Work in rat chondrocytes has shown that PGE2 can promote chondrocyte differentiation and may be an important contributor to cartilage formation (Miyamoto et al., 2003). In humans, synovial fluid concentrations of PGD2 are increased in people with established OA (Valdes et al., 2018), suggesting different functional roles of the PGs at different developmental stages of OA.

Potential associations between circulating oxylipin concentrations and synovial hyperplasia were studied. At 4 weeks post‐surgery, there was a negative association between members of the HETEs and synovitis score. Plasma levels of 15‐HETE are elevated in people with symptomatic knee OA, compared with non‐arthritic controls (Attur et al., 2015). Differences between the murine model and human data might reflect species differences or the stage of the model studied. At 8 weeks post‐surgery, 5‐HETE, 12‐HpETE, the 8,9‐, 11,12‐, 14,15‐ EETs and maresin‐2 were negatively associated with synovitis score. The EETs are anti‐inflammatory mediators derived from AA downstream of the cytochrome P450 pathway (Liu et al., 2005; Node et al., 1999). Previously, we demonstrated that synovial fluid concentrations of the by‐product of EET hydrolysis, the dihydroeicosatrienoic acids, are associated with human OA joint pathology in later stage OA (Valdes et al., 2018). We have also reported that plasma concentrations of the EETs and DHETs are associated with pain in people with OA (Gowler et al., 2021). The EETs are anti‐nociceptive (Inceoglu et al., 2011), and recent work has highlighted the role of the EETs in modulating experimental OA pain (Gowler et al., 2021; McReynolds et al., 2019). Despite the correlations with synovitis, there were no significant correlations between circulating oxylipins and synovial expression of the macrophage markers CD68 and CD206, suggesting contributions from other cell populations.

OA‐like pain behaviour was established at 16 weeks post‐surgery, and higher levels of 17‐HDHA were associated with less weight‐bearing asymmetry, indicative of less pain on loading. 17‐HDHA is a precursor of the D‐series resolvins, which are pro‐resolution mediators that promote the curtailing of inflammatory processes (Serhan et al., 2000). These data are in concordance with our previous report that increased circulating levels of 17‐HDHA are associated with lower pain responses in people with OA (Valdes et al., 2017). These changes in levels of 17‐HDHA are likely to have biological significance as exogenous systemic administration of 17‐HDHA has robust analgesic effects in rodent models of OA pain, strengthening the support for a key role of the resolvins in reducing OA pain (Huang et al., 2017). Interestingly, circulating 17‐HDHA was only related to changes in weight‐bearing, and not to changes in ipsilateral PWTs, which are partly mediated by changes in the spinal processing of nociceptive inputs. These findings may suggest a predominant modulation of peripheral nociceptive pathways by 17‐HDHA. This relationship between 17‐HDHA and pain at 16 weeks coincided with the significant increase in CD206 macrophage labelling observed in the synovia, which may be indicative of a switch to a pro‐resolution response at this time‐point.

Study limitations include that our measurements of synovial hyperplasia and macrophage infiltration had limited sensitivity. More sensitive methods may detect increases at earlier time‐points (Sambamurthy et al., 2018). However, previous reports also demonstrate that synovial macrophage expression was not altered at 8 weeks post‐DMM (Utomo et al., 2020). Further study of alternative markers for distinct macrophage populations, such as F4/80, CD163, iNOS, could provide in the future further detail on the cellular landscape of the synovia in the DMM model. The numbers of samples needed to assess differences in lipid concentrations in clinical samples exceeded those which could be feasibly used in an experimental model, we, therefore, focussed on the relationship between lipid concentrations, and OA pain and pathology. Although the extent to which concentrations of lipid concentrations measured in plasma reflect those in the knee joint needs further exploration, significant correlations between sera and synovial fluid concentrations of some of the ω‐3/ω‐6 PUFAs has been reported in the DMM model (Wu et al., 2017), suggesting at least partial concordance. The rationale for the use of male mice was the published variability in the progression of the DMM model in female C57BL/6 mice, and evidence this model is more robust in males (Ma et al., 2007; Malfait et al., 2010; von Loga et al., 2020), although comparable pain behaviours between male and female DMM mice have been reported (von Loga et al., 2020). We recognize the value of a comparative study in female mice, especially considering sex differences in neuroimmune contributions to chronic pain (reviewed in Mogil, 2020), however, the long timelines for the model necessitates this is undertaken in the future.

The results of these studies highlight the changing relationship between circulating lipid mediators, inflammatory synovial cell expression, OA pain and pathology at key time‐points in the development of OA. Using a slow‐progressing model of OA afforded the benefit of investigating changes during the early stages of OA, which are difficult to investigate clinically. Observing the changing relationship of the circulating lipids with cartilage damage and synovitis provides evidence that factors in the blood may reflect changes in the joint. Importantly, we saw that higher plasma concentrations of 17‐HDHA were related to less pain on loading, supporting previous clinical findings (Valdes et al., 2017) and validating the murine DMM model as a tool to explore the 17‐HDHA pathway as a therapeutic target for OA pain. Our demonstration that established pain was concurrent with an increase in macrophage expression in the synovia highlights the potential importance of synovitis in mediating joint pain. Further interrogation of the role of these oxylipin pathways in mediating OA pain may reveal novel analgesic targets.

## FUNDING INFORMATION

This work was funded by Versus Arthritis (Grant Number: 20777) and by the NIHR Nottingham Biomedical Research Centre.

## CONFLICT OF INTEREST

The authors declare no conflicts of interest.

## Supporting information


Table S1

Table S2

Table S3

Figure S1

Figure S2
