# Supplementary material for: Interplay between cellular changes in the knee joint, circulating lipids and pain behaviours in a slowly progressing murine model of osteoarthritis
Source: Eur J Pain. 2022 Sep 19;26(10):2213–26. doi: 10.1002/ejp.2036 (PMC9826505; doi:10.1002/ejp.2036)
Supplement: Supplementary file 1 — Table S1 [file EJP-26-2213-s001.docx]

| Cartilage Damage | Scoring Criteria |
| --- | --- |
| 0 | Normal |
| 1 | Small fibrillations without the loss of surface cartilage |
| 2 | Vertical clefts down to the layer immediately below the superficial layer and some loss of surface lamina |
| 3 | Vertical clefts to the calcified cartilage extending to <25% of the articular surface |
| 4 | Vertical clefts to the calcified cartilage extending to 25-50% of the articular surface |
| 5 | Vertical clefts to the calcified cartilage extending to >50-75% of the articular surface |
| 6 | Vertical clefts to the calcified cartilage extending to >75% of the articular surface |
|  |  |
| Synovitis | **Scoring Criteria** |
| 0 | Synovial lining 1 cell thick |
| 1 | Mild: Synovial lining 2-3 cells thick |
| 2 | Moderate: Synovial lining 4-5 cells thick |
| 3 | Severe: Synovial lining >6 cells thick |
|  |  |
| Chondrocyte Hypertrophy | **Scoring Criteria** |
| 0 | No chondrocyte hypertrophy in the non-calcified cartilage |
| 1 | Enlarged chondrocyte lacunae with lack of stain around a collapsed cell in the non-calcified cartilage |
|  |  |
|  |  |
| Osteophyte Score | **Scoring Criteria** |
| 0 | None |
| 1 | Small, same thickness as the adjacent cartilage |
| 2 | Medium, 1-3x the thickness as the adjacent cartilage |
| 3 | Large, >3x the thickness as the adjacent cartilage |
|  |  |
| Osteophyte Maturity | **Scoring Criteria** |
| 0 | None |
| 1 | Predominantly cartilaginous |
| 2 | Mixed cartilage and bone with active vascular invasion and endochondral ossification |
| 3 | Predominantly bone |
|  |  |
| Proteoglycan Loss | **Scoring Criteria** |
| 0 | Normal staining of non-calcified cartilage |
| 1 | Decreased but not complete loss of Saf-O staining over 1-100% of articular surface |
| 2 | Complete loss of Saf-O staining in the non-calcified cartilage extending to <25% of the articular surface |
| 3 | Complete loss of Saf-O staining in the non-calcified cartilage extending to >25-50% of the articular surface |
| 4 | Complete loss of Saf-O staining in the non-calcified cartilage extending to >50-75% of the articular surface |
| 5 | Complete loss of Saf-O staining in the non-calcified cartilage extending to >75% of the articular surface |
|  |  |
| Subchondral Bone Thickening | **Scoring Criteria** |
| 0 | Normal trabecular bone with >50% marrow space |
| 1 | 2 or more ‘wide’ vertical trabecular struts of bone that extend from the cartilage to the growth plate or ‘solid’ bone spanning up to 1/3^rd^ of the width of the epiphysis |
| 2 | ‘solid’ bone spanning up to >1/3^rd^ - <2/3^rd^ of the width of the epiphysis |
| 3 | ‘solid’ bone spanning up to >2/3^rd^ of the width of the epiphysis |
