# Supplementary material for: Interplay between cellular changes in the knee joint, circulating lipids and pain behaviours in a slowly progressing murine model of osteoarthritis
Source: Eur J Pain. 2022 Sep 19;26(10):2213–26. doi: 10.1002/ejp.2036 (PMC9826505; doi:10.1002/ejp.2036)
Supplement: Supplementary file 2 — Table S2 [file EJP-26-2213-s002.docx]

| **Lipid** | **Lipid plasma concentration nM ± SD** | | | | | | | | | | | | | | | | | |
| --- | --- | --- | --- | --- | --- | --- | --- | --- | --- | --- | --- | --- | --- | --- | --- | --- | --- | --- |
|  | **Sham Week 4** | | | **DMM Week 4** | | | **Sham Week 8** | | | **DMM Week 8** | | | **Sham Week 16** | | | **DMM Week 16** | | |
| **PGE2** | 1.00 | ± | 0.71 | 0.53 | ± | 0.21 | 0.53 | ± | 0.44 | 0.67 | ± | 0.45 | 0.69 | ± | 0.22 | 0.57 | ± | 0.33 |
| **PGD2** | 1.46 | ± | 0.53 | 1.10 | ± | 0.34 | 0.98 | ± | 0.19 | 1.24 | ± | 0.50 | 1.23 | ± | 0.26 | 1.08 | ± | 0.43 |
| **TBX B2** | 41.94 | ± | 39.81 | 26.74 | ± | 19.28 | 88.36 | ± | 178.01 | 31.29 | ± | 42.36 | 53.63 | ± | 44.13 | 42.13 | ± | 29.30 |
| **LTB4** | 6.66 | ± | 3.91 | 5.59 | ± | 3.09 | 4.18 | ± | 2.20 | 4.11 | ± | 2.94 | 4.81 | ± | 2.80 | 6.52 | ± | 3.92 |
| **6-T-LTB4** | 1.04 | ± | 0.35 | 0.81 | ± | 0.24 | 0.48 | ± | 0.31 | 0.84 | ± | 1.09 | 0.78 | ± | 0.51 | 0.96 | ± | 0.69 |
| **5-HETE** | 27.53 | ± | 12.73 | 21.59 | ± | 9.14 | 15.99 | ± | 6.91 | 16.75 | ± | 10.16 | 19.67 | ± | 7.22 | 25.00 | ± | 15.34 |
| **8-HETE** | 11.62 | ± | 1.92 | 9.20 | ± | 3.53 | 8.68 | ± | 3.26 | 10.24 | ± | 3.86 | 10.25 | ± | 2.47 | 10.85 | ± | 4.13 |
| **9-HETE** | 3.80 | ± | 2.08 | 2.59 | ± | 0.88 | 3.21 | ± | 2.15 | 3.28 | ± | 2.52 | 3.51 | ± | 2.38 | 2.72 | ± | 1.32 |
| **11-HETE** | 2.55 | ± | 1.97 | 1.32 | ± | 0.83 | 1.95 | ± | 1.80 | 1.94 | ± | 2.96 | 1.58 | ± | 0.97 | 1.63 | ± | 1.30 |
| **12-HETE** | 839.37 | ± | 374.00 | 691.60 | ± | 423.51 | 528.51 | ± | 215.39 | 763.11 | ± | 364.32 | 723.83 | ± | 314.82 | 839.13 | ± | 398.34 |
| **15-HETE** | 23.63 | ± | 11.10 | 18.14 | ± | 6.82 | 17.92 | ± | 10.24 | 20.60 | ± | 11.87 | 24.75 | ± | 8.00 | 21.28 | ± | 9.13 |
| **5-HpETE** | 13.91 | ± | 3.50 | 13.32 | ± | 4.52 | 15.76 | ± | 7.14 | 18.15 | ± | 8.29 | 28.78 | ± | 37.32 | 12.44 | ± | 1.00 |
| **12-HpETE** | 13.86 | ± | 3.82 | 12.11 | ± | 3.16 | 12.03 | ± | 2.54 | 15.90 | ± | 7.97 | 22.37 | ± | 26.51 | 13.94 | ± | 5.29 |
| **5,6-EET** | 15.44 | ± | 8.67 | 13.25 | ± | 8.89 | 14.59 | ± | 7.77 | 28.06 | ± | 48.20 | 21.95 | ± | 13.95 | 23.03 | ± | 13.45 |
| **8,9-EET** | 2.00 | ± | 1.21 | 1.64 | ± | 0.68 | 2.97 | ± | 3.62 | 2.25 | ± | 2.60 | 2.11 | ± | 0.83 | 2.44 | ± | 1.65 |
| **11,12-EET** | 5.93 | ± | 2.90 | 4.21 | ± | 1.53 | 5.11 | ± | 3.95 | 4.76 | ± | 4.04 | 4.83 | ± | 2.19 | 5.03 | ± | 2.89 |
| **14,15-EET** | 6.53 | ± | 2.88 | 4.94 | ± | 1.76 | 5.77 | ± | 3.34 | 6.03 | ± | 5.09 | 6.51 | ± | 2.29 | 6.22 | ± | 3.00 |
| **11,12-DHET** | 2.79 | ± | 1.95 | 2.66 | ± | 1.25 | 2.28 | ± | 1.63 | 1.96 | ± | 0.55 | 2.72 | ± | 0.95 | 3.59 | ± | 2.52 |
| **14,15-DHET** | 4.91 | ± | 3.49 | 4.54 | ± | 1.96 | 4.59 | ± | 4.46 | 3.62 | ± | 0.95 | 5.27 | ± | 2.54 | 6.06 | ± | 4.08 |
| **9-HODE** | 66.53 | ± | 22.80 | 47.68 | ± | 21.35 | 72.20 | ± | 71.74 | 65.00 | ± | 32.80 | 73.32 | ± | 17.73 | 83.41 | ± | 39.08 |
| **13-HODE** | 169.83 | ± | 38.90 | 136.62 | ± | 65.76 | 151.02 | ± | 136.06 | 159.73 | ± | 47.64 | 173.07 | ± | 42.76 | 180.88 | ± | 90.20 |
| **9-OxoODE** | 26.99 | ± | 6.16 | 16.97 | ± | 8.99 | 36.22 | ± | 56.81 | 24.93 | ± | 19.21 | 26.05 | ± | 8.32 | 25.34 | ± | 14.92 |
| **13-OxoODE** | 37.88 | ± | 8.79 | 28.50 | ± | 8.96 | 45.40 | ± | 45.04 | 39.69 | ± | 22.23 | 40.37 | ± | 17.62 | 43.76 | ± | 15.18 |
| **5,12-DiHETE** | 4.13 | ± | 1.90 | 3.27 | ± | 2.52 | 1.38 | ± | 0.68 | 4.19 | ± | 7.19 | 2.55 | ± | 1.84 | 2.76 | ± | 2.13 |
| **5,15-DiHETE** | 0.27 | ± | 0.13 | 0.24 | ± | 0.14 | 0.18 | ± | 0.07 | 0.26 | ± | 0.27 | 0.29 | ± | 0.25 | 0.21 | ± | 0.12 |
| **8,9-DiHETE** | 0.24 | ± | 0.05 | 0.23 | ± | 0.14 | 0.16 | ± | 0.06 | 0.32 | ± | 0.43 | 0.32 | ± | 0.15 | 0.31 | ± | 0.18 |
| **17-HDHA** | 7.65 | ± | 2.17 | 5.87 | ± | 2.10 | 7.29 | ± | 5.97 | 8.04 | ± | 3.93 | 7.90 | ± | 2.26 | 6.55 | ± | 2.91 |
| **RvD5** | 0.34 | ± | 0.10 | 0.32 | ± | 0.11 | 0.23 | ± | 0.17 | 0.34 | ± | 0.17 | 0.32 | ± | 0.13 | 0.24 | ± | 0.10 |
| **Maresin 2** | 1.11 | ± | 0.43 | 0.98 | ± | 0.36 | 0.82 | ± | 0.24 | 1.45 | ± | 1.37 | 1.52 | ± | 1.57 | 0.88 | ± | 0.30 |
| **2-AG** | 308.45 | ± | 76.90 | 281.84 | ± | 102.39 | 209.54 | ± | 93.78 | 220.67 | ± | 66.90 | 324.95 | ± | 105.11 | 299.73 | ± | 101.80 |
| **AEA** | 0.54 | ± | 0.10 | 0.53 | ± | 0.07 | 0.60 | ± | 0.15 | 0.89 | ± | 0.60 | 0.57 | ± | 1.27 | 0.60 | ± | 0.18 |
| **OEA** | 5.24 | ± | 0.96 | 5.23 | ± | 0.87 | 7.10 | ± | 8.53 | 5.23 | ± | 1.32 | 7.66 | ± | 8.78 | 6.01 | ± | 1.89 |
| **PEA** | 9.14 | ± | 2.95 | 8.31 | ± | 2.36 | 16.03 | ± | 24.27 | 7.61 | ± | 2.11 | 8.18 | ± | 1.96 | 10.37 | ± | 3.71 |
