# Supplementary material for: Interplay between cellular changes in the knee joint, circulating lipids and pain behaviours in a slowly progressing murine model of osteoarthritis
Source: Eur J Pain. 2022 Sep 19;26(10):2213–26. doi: 10.1002/ejp.2036 (PMC9826505; doi:10.1002/ejp.2036)
Supplement: Supplementary file 3 — Table S3 [file EJP-26-2213-s004.docx]

|  |  |  | **Week 4** | |  | **Week 8** | |  | **Week 16** | |
| --- | --- | --- | --- | --- | --- | --- | --- | --- | --- | --- |
|  | **Lipid** |  | **P - Value** | **R Value** |  | **P - Value** | **R - Value** |  | **P - Value** | **R - Value** |
|  |  |  |  |  |  |  |  |  |  |  |
| **Cartilage Damage** | PGE2 |  | **0.006** | **-0.670** |  | 0.491 | 0.192 |  | 0.557 | -0.148 |
|  | PGD2 |  | **0.035** | -0.518 |  | 0.577 | -0.155 |  | 0.124 | -0.388 |
|  | 5-HETE |  | 0.159 | -0.357 |  | **0.049** | -0.519 |  | 0.820 | -0.059 |
|  | 8-HETE |  | **0.036** | **-0.517** |  | 0.243 | -0.320 |  | 0.915 | -0.027 |
|  | 12-HETE |  | **0.010** | **-0.617** |  | 0.689 | -0.112 |  | 0.882 | 0.038 |
|  | 15-HETE |  | 0.060 | -0.458 |  | 0.853 | -0.052 |  | 0.618 | -0.126 |
|  | 9-oxoODE |  | 0.774 | -0.078 |  | 0.573 | -0.157 |  | **0.004** | -0.686 |
|  | 17-HDHA |  | 0.418 | -0.208 |  | 0.346 | -0.260 |  | 0.066 | -0.441 |
|  |  |  |  |  |  |  |  |  |  |  |
|  |  |  |  |  |  |  |  |  |  |  |
| **Synovitis** | 5-HETE |  | **0.025** | **-0.546** |  | **0.043** | **-0.516** |  | 0.892 | -0.036 |
|  | 8-HETE |  | 0.069 | -0.453 |  | 0.138 | -0.388 |  | 0.372 | 0.223 |
|  | 11-HETE |  | 0.836 | -0.056 |  | **0.011** | **-0.634** |  | 0.369 | 0.239 |
|  | 12-HETE |  | 0.099 | -0.414 |  | 0.531 | -0.168 |  | 0.567 | 0.144 |
|  | 12-HpETE |  | 0.663 | 0.113 |  | **0.009** | **-0.640** |  | 0.393 | 0.214 |
|  | Resolvin D5 |  | 0.710 | 0.109 |  | 0.055 | -0.632 |  | 0.977 | -0.009 |
|  | Maresin 2 |  | 0.324 | 0.262 |  | 0.064 | -0.511 |  | 0.721 | -0.093 |
|  |  |  |  |  |  |  |  |  |  |  |
|  |  |  |  |  |  |  |  |  |  |  |
| **Pain Behaviour** | 17-HDHA | %WB | 0.362 | 0.228 |  | 0.460 | -0.186 |  | **0.047** | **0.448** |
|  |  | VF | 0.074 | 0.432 |  | 0.816 | -0.059 |  | 0.602 | 0.124 |
|  | Resolvin D5 | %WB | 0.361 | 0.254 |  | 0.869 | -0.056 |  | 0.091 | 0.424 |
|  |  | VF | 0.612 | 0.141 |  | 0.943 | -0.025 |  | 0.699 | 0.101 |
