# Supplementary figures and images for: Interplay between cellular changes in the knee joint, circulating lipids and pain behaviours in a slowly progressing murine model of osteoarthritis
Source: Eur J Pain. 2022 Sep 19;26(10):2213–26. doi: 10.1002/ejp.2036 (PMC9826505; doi:10.1002/ejp.2036)

**A**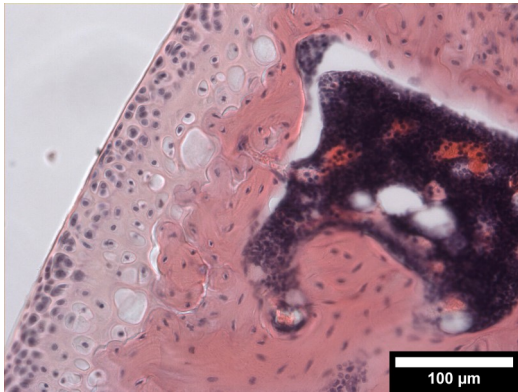**B**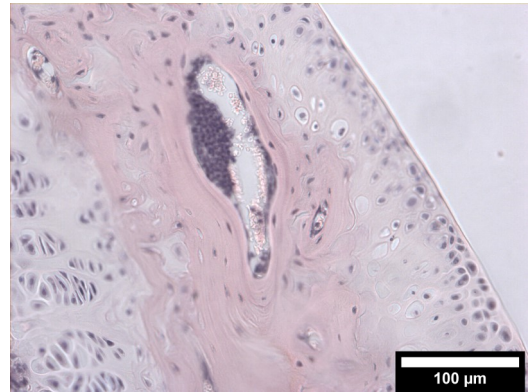**C**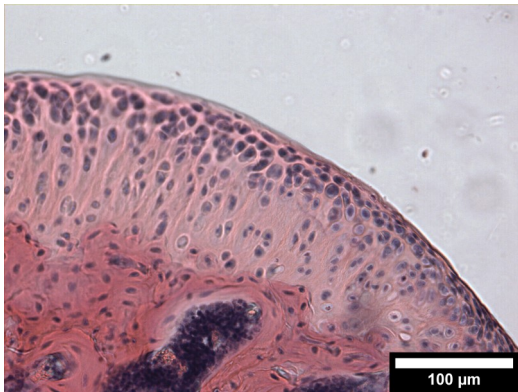**D**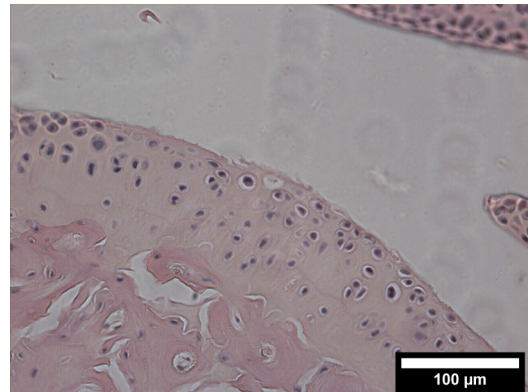**E**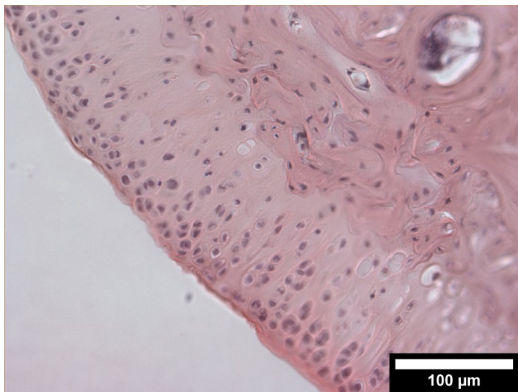**F**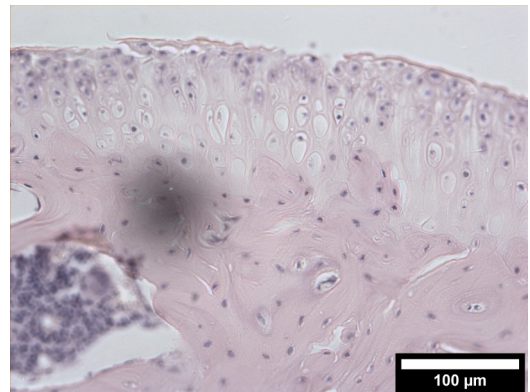

Supplement: Supplementary file 4 — Figure S1 [file EJP-26-2213-s005.pdf]

# Week 16

## Cartilage Damage

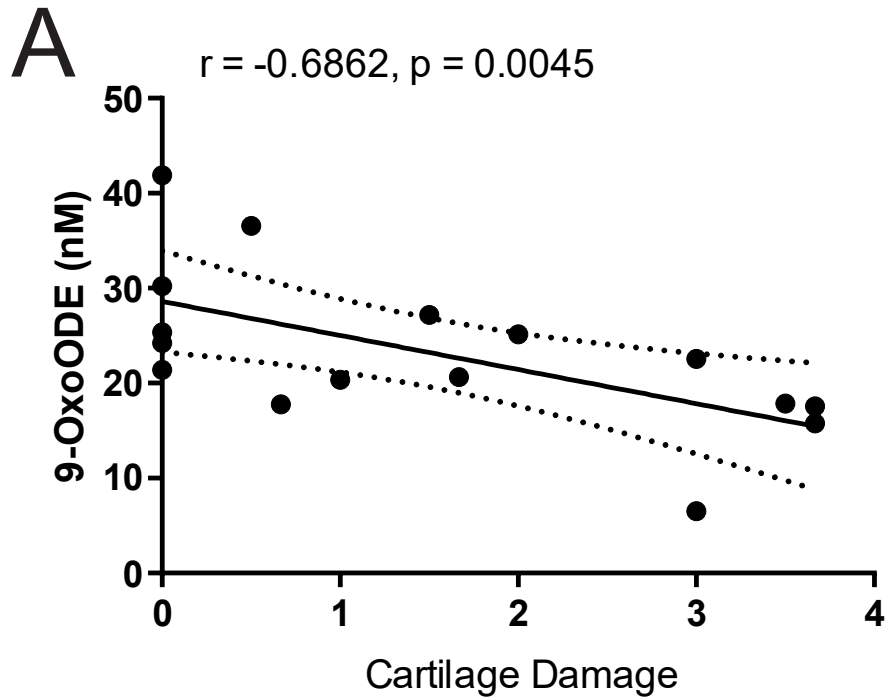

Supplement: Supplementary file 5 — Figure S2 [file EJP-26-2213-s003.pdf]
